# Cherry-Pit Perforation of an Ileocecal Diverticulum: A Case Report

**DOI:** 10.7759/cureus.87312

**Published:** 2025-07-05

**Authors:** Yoichi Miyaoka, Shingo Shimada, Kazuhiro Ogasawara, Akinobu Taketomi

**Affiliations:** 1 General Surgery, Abashiri-Kosei General Hospital, Abashiri, JPN; 2 Surgery, Otaru General Hospital, Otaru, JPN; 3 Surgery, Kushiro Rosai Hospital, Kushiro, JPN; 4 Gastroenterological Surgery 1, Hokkaido University Graduate School of Medicine, Sapporo, JPN

**Keywords:** cherry pit, colonic perforation, foreign body, fruit-seed gastrointestinal injury, ileocecal diverticulum

## Abstract

Seed-induced colonic perforation is exceptionally rare but may mimic malignancy or complicated diverticulitis, leading to diagnostic challenges. We report the case of a middle-aged man who presented with acute right lower quadrant abdominal pain. Contrast-enhanced computed tomography (CT) revealed mural thickening and a target-like lesion at the ileocecal junction, raising suspicion for intussusception or carcinoma. Emergency surgery identified a cherry pit perforating a pseudodiverticulum in the ileocecal region. This case highlights the potential for hard fruit seeds to cause full-thickness bowel injury, especially in right-sided pseudodiverticula, which are more prevalent in Asian populations and lack a protective muscularis layer. Characteristic imaging features, such as hyperdense intraluminal objects with surrounding inflammation, can assist in differentiating these cases from neoplastic or inflammatory conditions. Taking a detailed dietary history and carefully reviewing CT scans using bone window settings can facilitate early diagnosis. Clinicians should remain aware of this rare but serious entity, as prompt surgical intervention is crucial in the presence of perforation or generalized peritonitis.

## Introduction

Foreign-body ingestion is common, yet less than 1% of objects cause perforation [1]. Fish bones dominate such injuries, whereas fruit seeds are rarely implicated. Seeds become hazardous when they lodge at anatomical narrows or within diverticula and are exposed to propulsive peristalsis [2,3]. The sigmoid colon is classically affected, but right-sided lesions are increasingly recognized in regions with prevalent right-colon diverticulosis. Notably, the prevalence of colonic diverticulosis varies by geography: while left-sided diverticula are more common in Western populations, right-sided diverticula predominate in East Asian countries, where prevalence among adults can exceed 20% [4,5]. We present a case of cherry-pit perforation of an ileocecal diverticulum and summarize recently reported seed-related large-bowel complications.

## Case presentation

A 46-year-old man with chronic low back pain managed by regular nonsteroidal anti-inflammatory drugs (NSAIDs) and no history of abdominal surgery presented with a two-day history of progressively worsening colicky pain in the right iliac fossa. The pain persisted despite his ingestion of a greater-than-usual dose of NSAIDs. He denied nausea and vomiting. On arrival at our hospital, his vital signs were stable. Abdominal examination revealed marked tenderness with rebound in the right lower quadrant. Laboratory investigations showed a white-blood-cell count of 21 × 10³/µL and a C-reactive protein level of 9.12 mg/dL.

**Figure 1:**
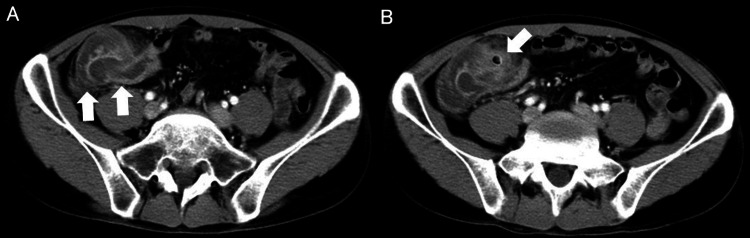
Abdominal CT. (A) Contrast-enhanced CT shows ileocolic intussusception with concentric mural thickening of the terminal ileum and cecum and surrounding inflammatory fat stranding (arrows). (B) An intraluminal structure with a centrally hypoattenuating core and hyperattenuating rim is visible (arrow) CT: computed tomography

Contrast-enhanced abdominal computed tomography (CT) revealed concentric mural thickening at the ileocecal junction, surrounding fat stranding, and a target configuration consistent with intussusception. A discrete intraluminal hyperdense focus, slightly denser than bowel contents, was present but not definitively characterized or diagnosed (Figure 1).

**Figure 2:**
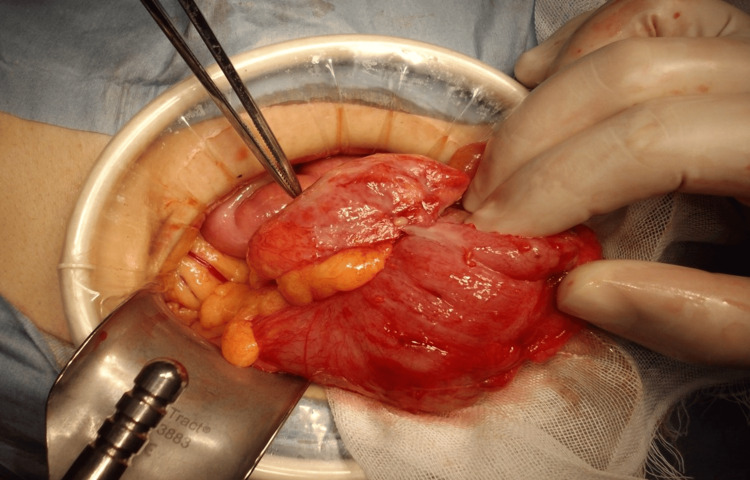
Intraoperative photograph demonstrating marked inflammation and adhesions around the cecum, with telescoping (intussusception) of the adjacent bowel

Emergency midline laparotomy uncovered dense inflammatory adhesions around the cecum (Figure 2).

**Figure 3:**
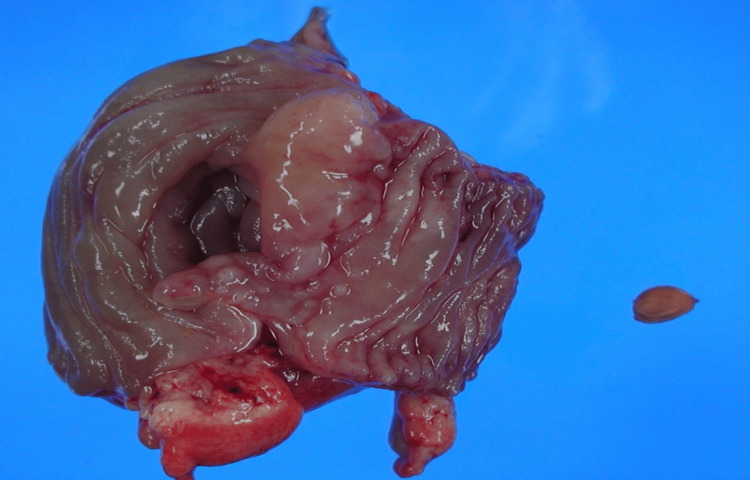
Resected ileocecal specimen with a 13 × 10 mm cherry pit lodged in a perforated pseudodiverticulum and surrounding acute serositis

Several small pseudodiverticula were identified on the antimesenteric border; one diverticulum was perforated by an oval brown cherry pit measuring 13 × 10 mm, which extended into the mesentery (Figure 3).

An ileocecal resection with side-to-side stapled anastomosis was completed. Oral intake was resumed on postoperative day 3, and the patient was discharged on day 5. Postoperative dietary questioning revealed that he had swallowed whole cherries one week before admission.

## Discussion

This case underscores that even a small cherry pit can precipitate a life-threatening perforation of the right colon. Three pathophysiologic mechanisms appear to be involved. First, the pit’s rigid, lignin-rich shell withstands enzymatic degradation, and its microscopic ridges mechanically abrade the intestinal mucosa [6]. Second, after dehydration, the pit absorbs intraluminal fluid and swells in situ; its rough surface facilitates bacterial adherence, thereby amplifying local inflammation and ischemia [2,7]. Third, once the impacted pit lodges within a false diverticulum lacking muscularis propria, peristaltic forces exert focal pressure that can culminate in full-thickness necrosis and perforation [8].

Recent case reports (Table 1) [6,9-13] indicate that seed-related colonic complications can also arise in the right colon, particularly in Asian populations. This predominance reflects both the high prevalence of cecal and ascending colon diverticulosis in these populations and regional dietary habits that include the consumption of seed-containing fruits. Although the right colon has a wider lumen, its thinner wall can rapidly fail when a hard seed becomes impacted in a false diverticulum; peristaltic compression then produces focal necrosis and full-thickness perforation, explaining the dramatic presentations observed.

**Table 1:** Seed-related terminal ileum-ascending colon complications NR: not reported; POD: postoperative day

Year	Study	Seed/pit	Site	Presentation → management	Outcome	Age/sex	Country
1999	Fujikawa et al. [6]	Pickled-plum seed	Cecum	Perforated peritonitis → segmental colectomy	Survived	NR	Japan
2005	Otani et al. [9]	Persimmon × 5	Cecum ＋ ascending colon	Large-bowel obstruction → emergency R-hemicolectomy	Discharged POD 26	82 years/male	Japan
2006	Ashida et al. [10]	Pickled-plum seed	Ascending-colon cancer ulcer	Ileus → right hemicolectomy	Uneventful	78 years/female	Japan
2008	Yamaguchi et al. [11]	Watermelon and corn seeds (multiple)	Ascending colon	Seed-bezoar ileus → right hemicolectomy	Discharged POD 23	71 years/male	Japan
2011	Puia et al. [12]	Multiple fruit stones	Ascending colon	Perforation → right hemicolectomy	Uneventful	Two cases/NR	Romania
2016	Gupta et al. [13]	Plum seed	Terminal ileum	Perforation in hernia → ileocecal resection	Well	11 months/male	India
2025	Present case	Cherry pit	Ileocecal	Ileocecal perforation → ileocecal resection	Discharged POD 5	46 years/male	Japan

Fruit seeds may exhibit mixed attenuation on CT, with the dense outer shell appearing hyperattenuating (approximately 100-300 Hounsfield units) and the hollow or pulp-deficient core appearing hypoattenuating. Such findings can be mistaken for stool or contrast material, but bone window settings and multiplanar reconstructions aid in accurate identification [14]. Host factors also modulate risk: in this case, the patient had been taking NSAIDs regularly. Previous reports, including a case of a solitary cecal ulcer that resolved after discontinuation of loxoprofen [15], as well as several cases of ileocecal ulceration or stenosis associated with long-term NSAID use [16], suggest that chronic loxoprofen therapy can compromise mucosal integrity in the terminal ileum to the ascending colon. Therefore, it is possible that long-term loxoprofen use in this patient contributed to intestinal wall fragility, facilitating seed impaction and subsequent perforation. Therefore, chronic NSAID therapy, impaired mastication, and consuming fruits without removing their seeds are risk factors for seed impaction that can lead to obstruction or perforation in patients with right-sided colonic diverticulosis, especially in populations where large fruit pits or seeds are commonly eaten.

## Conclusions

Cherry pits lodged in the ileocecal pseudodiverticula can cause acute perforation that mimics intussusception or carcinoma. Accurate CT interpretation and prompt surgery are pivotal for favorable outcomes; in particular, clinicians should remain vigilant for seed-related perforations throughout the colon, especially in regions where right-sided diverticulosis predominates.
